# Case report: lymphoepithelial-like carcinoma of the lung-a chronic disease?

**DOI:** 10.1186/1477-7819-10-91

**Published:** 2012-05-21

**Authors:** Joelle FS Wong, Melissa CC Teo

**Affiliations:** 1 Department of Surgical Oncology, National Cancer Centre, 11 Singapore General Hospital, Outram Road, Singapore, 169608, Singapore

**Keywords:** Lymphoepithelial-like carcinoma, Non small cell lung cancer, Epstein-barr virus, Metastatic lung cancer

## Abstract

This is a case of metastatic lung cancer of the lymphoepithelial-like carcinoma (LELC) variant who first presented with symptomatic brain metastasis. The patient underwent local and systemic treatment for metastatic disease with good clinical outcome. The patient was disease free for four years then she had primary lung recurrence which was surgically resected. She underwent a second course of chemotherapy with saw her through another two years of disease free period. A recurrence of the cancer was detected intra-abdominally on the seventh year of diagnosis. This was treated again with surgical resection and another course of chemotherapy.

## Background

We report a case of primary non-small cell lung cancer of the lymphoepithelioma variant in a woman who presented with symptomatic brain metastasis. She underwent initial treatment with chemo-radiation therapy to her brain metastasis and primary lung tumor. Post treatment, she enjoyed a significant disease free interval of 4 years but returned with local recurrence in the lungs which was treated with local resection. She remained disease-free on surveillance until 2 years later when a new soft tissue mass was detected at the retroperitoneal/ posterior mediastinal region. The patient underwent a second operation to resect the new lesion. Histology again confirmed lymphoepithelioma-like carcinoma consistent with metastatic disease. Currently she is disease-free after two major surgeries, two sessions of radiotherapy and 3 courses of chemotherapy, 8 years post initial diagnosis of metastatic cancer.

## Case presentation

LGH, a 52 year old Chinese female, initially presentated with generalized seizures with no other complaints or symptoms in 2003. She had three separate episodes of generalized seizures over the course of three months before presenting at the hospital. Her attacks were witnessed by family and was described as a sudden loss of consciousness lasting approximately 10 minutes and associated with drooling and mild tonic phase with spontaneous recovery.

Clinically, she had no focal neurological deficit, cognitive dysfunction or visual disturbances. Her other systems review was also unremarkable. A magnetic resonance imaging (MRI) scan of the brain showed a mass in the left parieto-occipital lobe, in the parasagittal location with mixed solid cystic component measuring approximately 3.5x2.4x2.1 cm with surrounding vasogenic oedema. However there was no significant mass effect to cause ventricular deviation or cerebral herniation. The impression was that of metastatic brain tumour.

**Figure 1:**
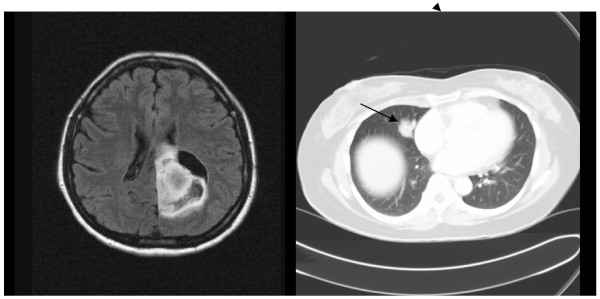
Pulmonary LELC in right lung with brain metastasis seen in left parieto-occipital lobe with localized mass effect and surrounding vasogenic oedema.

Computer tomographic (CT) scans of the thorax, abdomen and pelvis was performed to investigate for a primary lesion. The scans showed a 2.3x 2.3 cm lesion localized in the right lung as the only significant abnormal lesion. (Figure 1).

A transthoracic needle aspiration of the lung lesion confirmed the tumour to be an undifferentiated carcinoma of the lungs favouring a lymphoepithelial-like carcinoma. The tumour stained positively for Epstein-barr virus (EBV) on in-situ hybridization (ISH) studies. A computer tomographic (CT) scan of the postnasal space done showed no abnormality or other evidence to suggest a primary EBV-related nasopharyngeal or salivary gland type carcinoma. Nasal endoscopy was also performed by the otolaryngologist and was negative for suspicious nasopharyngeal mucosal lesion or tumour. Further review of the scans did not show significant lymphadenopathy to implicate lymphoma (Burkitt, Hodgkin’s and non-Hodgkin’s) as the pathogenesis of malignancy.

Her case was discussed at a multidisciplinary tumour board meeting. The treatment plan decided by the panel was first to treat her symptoms of seizures with targeted radiotherapy to her brain metastasis, followed by targeted treatment for her primary lung carcinoma. Treatment goals were to control disease related symptoms and to prolong survival.

She underwent 13 days of whole brain radiotherapy (WBRT) at 30 Gy as initial treatment with minimum side effects. Her seizures were well controlled by oral anti-epileptic drug (phenytoin) with no occurrence of seizure during and after the course of radiotherapy.

Her post treatment scans showed minimal post radiotherapy oedema that was managed well with corticosteroids. There was no evidence of widespread disease within the brain post WBRT. Her bone scan showed no evidence of metastatic bone disease at this stage. In view of her response to the initial therapy and her good performance status, a chemo-radio therapeutic regimen was planned for treatment of the primary lung lesion.

A Platinum-based chemotherapy regimen (Paclitaxel/ Carboplatin) was given concurrently with a total dose of 60 Gy radiation therapy in 30 cycles divided into 2 phases, completed in 6 weeks. Using stereotatic radiotherapy techniques, the external treatment radiation beam was directed to conform to the shape of the lung tumor, hence minimizing the toxicity of radiation to the surounding tissue.

**Figure 2:**
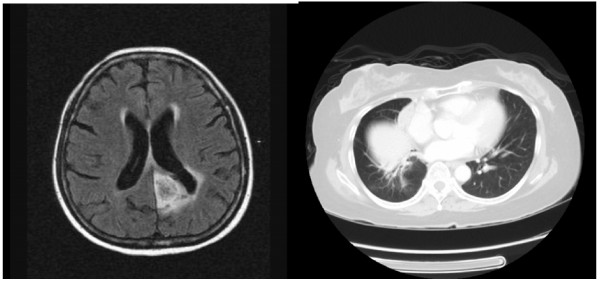
**Pulmonary LELC with brain metastasis after initial radio and chemotherapy.** Size of brain metastasis is smaller with decrease in surrounding oedema and mass effect.

She remained well on follow-up with no evidence of recurrence on surveillance scans (Figure 2). Post treatment, she developed delayed drug hypersensitivity syndrome to phenytoin. This was managed with oral and topical corticosteriods and her seizures remained well controlled with the change of her anti-epileptic drug to Levetiracitam.

**Figure 3:**
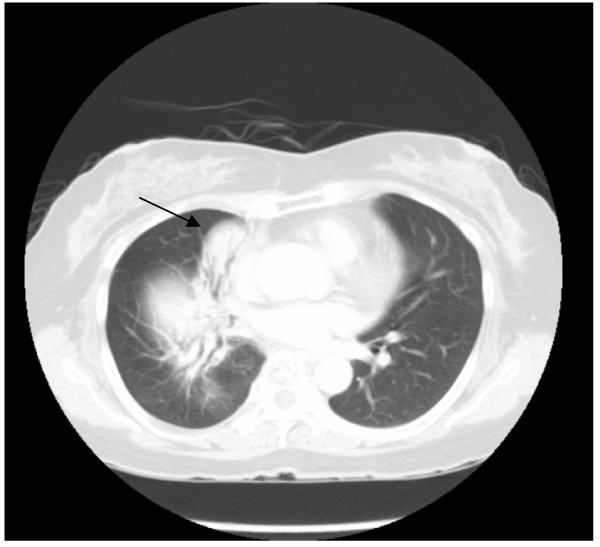
Pulmonary LELC with local recurrence in right lung (pre-resection).

LGH continued with regular surveillance follow-up with no evidence of recurrence on surveillance CT brain, thorax, abdomen and pelvis for 4 years post treatment. In the 5^th^ year, her surveillance scans showed a new local recurrence in the lung (Figure 3). The decision was made for surgical resection of the local recurrence, considering the resectability of the tumor and the fact that she had already received full-dose chemoradiation treatment to the same anatomical region.

The patient underwent a right middle lobectomy. The resected tumor was well confined with a microscopic dimension of 3 cm. The pleura, bronchovascular and parenchymal margins were all free from tumor. A total of 17 benign lymph nodes were resected.

She recovered uneventfully from the thoracotomy. Post-operatively, she underwent four cycles of Platinum-based combination (Gemcitabine/ Carboplatin) chemotherapy.

**Figure 4:**
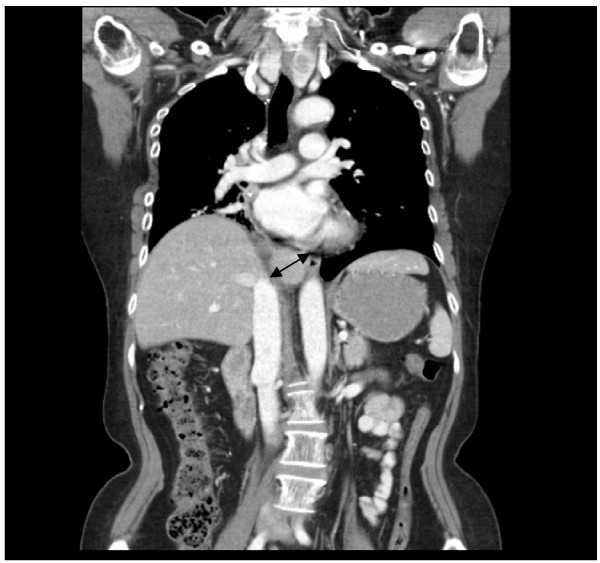
Soft tissue mass discovered at the retoperitoneal/ mediastinal region (pre-resection).

She remained disease-free for another 2 years. In her 7^th^ year post diagnosis, her surveillance scans revealed a 3x2.1 cm soft tissue mass seen at the retroperitoneal/ posterior mediastinal region. (Figure 4)

In view of her long disease free interval and good response to treatment, LGH was offered and proceeded to a resection of the new retroperitoneal soft tissue mass. During surgery, the mass was approached intraabdominally via an upper midline incision. Complete excision was made possible by dividing the arcuate ligament and widening the oesophageal hiatus, hence avoiding a mediastinotomy. The mass was found adjacent to foregut derivatives like the oesophagus and stomach but with no gross evidence of invasion. The diaphramatic defect was repaired and a right chest tube was inserted post operatively.

Her post operative recovery was unremarkable. The histology confirmed the tumour to be a metastasis of the primary lung lymphoepithelioma-like carcinoma. LGH went on to receive her third course of Platinum-based combination regimen (Docetaxel/ Carboplatin).

She remains well and disease-free six months after her most recent surgery and eight years from her initial presentation.

## Discussion

Lymphoepithelial-like carcinoma are uncommon cancers. They are usually found in pharyngeal and foregut derivatives like salivary glands, thymus, stomach and liver. However, recent cases of LELC detected in other anatomical locations were also reported, such as bladder [1], lacrimal glands [2], ovaries [3], cervix [4], skin [5] etc. Since the first reported case of LELC in the lower respiratory tract by Begin [6] et al, there has been approximately 150 reported cases of lung LELC worldwide in the literature.

Majority of the reported cases of LELC in current literature are stage III or lower. This is a case report of stage IV metastatic lymphoepithelioma-like carcinoma (LELC) of the lungs which presented with synchronous brain metastasis. It is unique as it illustrates a case of treated metastatic NSCLC that far exceeded her predicted life expectancy. With ongoing surveillance and treatment, selected advanced cancers can be managed like a chronic disease.

Histopathologically, LELC is similar to undifferentiated nasopharyngeal carcinoma (NPC). It is characterized by a diffused growth of epithelial cells on a background of inflammatory cells, predominantly lymphocytes. Lung LELCs are less common, accounting for only 0.15 [7] to 3.6% [8] of all lung carcinoma with most of the literature published on the Asian population. LELC of the lung has been shown to have an association with EBV infection. This is especially so in the Asian Chinese population but not the Caucasian population. LELCs of the bladder, skin, vagina, and cervix have no EBV association [9].

Case studies have shown association of silicosis with LELC of the lungs where occupational exposure to silica dust is implicated to result in carcinogenesis [10]. There is also an excess of lung cancer cases in silicosis registry. Although pathogenesis of carcinoma has been established in animal studies but human meta-analyses of the epidemiology studies have not been conclusive of this relationship [11,12]. It was however reported that silica-exposed workers have a relative risk of 1.3 and higher at 2.8 if workers are silicotic. Hence, it is recommended by the National Institute for Occupational Safety and Health (NIOSH) in the United States that crystalline silica be considered a potential occupational carcinogen [13].

Patients wih lung LELC are often 10 years younger than patients with other histological subtypes of NSLCs. Our patient from this reported case is a 52 year old Chinese female who fits the commonly reported LELC profile of a non-smoker who is of Asian descent close to the median age of 48 years old.

She first presented with symptoms suggestive of a space occupying lesion in the cranium causing generalized seizures. Primary brain tumours were considered as the initial diagnosis but metastatic brain tumors could not be ruled out. The most common primary malignancies linked to brain metastasis are lung, breast, skin and colonic. After a proper oncological work up, a lung primary with metastasis to the brain is identified.

When lung biopsies turn up positive for carcinoma, coupled with the identification of EBV infection, NPC primary with lung metastasis has to be excluded first. Differentiation in the diagnosis of primary lymphoepithelioma versus primary NPC with metastasis to lungs is necessary due to the different approach in management. Radiation is the mainstay of treatment in NPC. On the contrary, surgery is the major curative modality for NSCLC and in higher stage carcinomas, radio-chemotherapy is often planned post-operatively. Hence any mistaken histopathological conclusions made would result in improper cancer staging and patient management.

**Figure 5:**
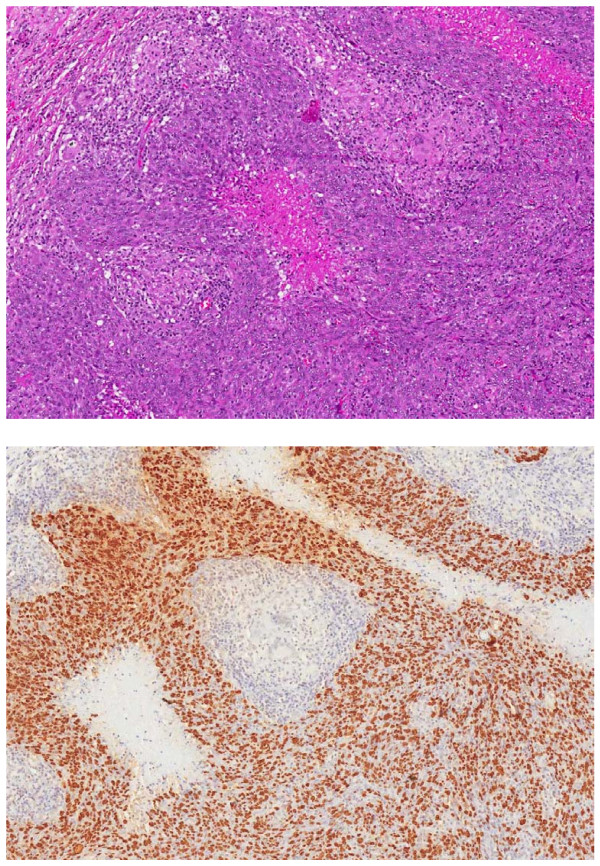
**Lung tissue from lobectomy showing malignant LELC cells (H&E stain x10) and positive EBV insitu hybridization (EBER – ISH x10) Stained brown.** Magnification x10.

Radiologically on CT, both primary lung cancer lesions and metastic NPC lung lesions are indistinguishable. Pathologically, both are similarly associated to EBV infection. Figure 5 This case highlights the importance of avoiding the diagnostic pitfall of assuming that the lung biopsy of a LELC points definitely to a primary carcinoma of the lung.

Early stage presentation of LELC can often be picked up on CT as poorly circumscribed, peripherally placed nodules <3.5 cm and not associated with lymphadenopathy [14,15]. The conclusion for advanced stage disease on the other hand showed features of large, well circumscribed, centrally located lesions with evidence of vascular encasement and peribronchovascular lymphadenopathy. These features are also similarly seen in primary bronchogenic carcinoma [16]. In our reported case of LELC presenting to us at stage IV disease with brain metastasis, the primary lung carcinoma on CT imaging was well-defined with lobulated margins and associated with ipsilateral hilar lymphadenopathy.

The characteristics of LELC on MRI are non-specific but are helpful in staging (eg. neural invasion) and preoperative planning.

Positron Emission Tomographic (PET) imaging provides an alternative non-invasive method of determining the malignant potential of solitary pulmonary nodule. PET/CT was significantly more sensitive (96% vs 81%; *p* < 0.05) and accurate (93% vs 85% *p* = 0.011) than CT alone in characterizing lung nodules [17]. However, further characterization and identification of the nodule still requires a multi-disciplinary approach with determination of histological sub-type, grading and TNM staging for more precise diagnosis and prognosis [18].

PET imaging was not used in our reported case. However, in the case reported by cheng et al, the LELC of the lung was picked up on CT but was not suspicious on PET scan done approximately two years after initial presentation, showing a standardized uptake value (SUV) of <2.5 (considered malignant >3.5 SUV) [17,19]. Hence PET imaging alone might be of limited benefit in the case of LELC of the lung.

The patient in this case was diagnosed with stage IV lung cancer and was treated aggressively with multimodality treatment options of surgery, chemotherapy and radiotherapy. First she underwent symptomatic radiotherapy for her brain metastasis with good results, then further courses of radiation and chemotherapy to the primary lung cancer site. Surgery was offered to her when she returned after an interval of disease free period of five years with local recurrence of her lung primary. She recovered well from the thoracotomy. In view of patient’s complete resection of the primary lung tumour with no lymph node involvement, adjuvant chemotherapy was again offered to her. According to the new WHO classification of lung tumours [20], lymphoepithelioma-like carcinoma is recognised as a variant subtype of large cell carcinoma of the lung. The guidelines [21] recommend the use of adjuvant chemotherapy in patients with stages IIA/B to IIIA NSCLC in which the tumours have been completely resected for increase survival benefits. Hence after a second course of platinum-based chemotherapy, her survival was prolonged by another two years.

Prognosis studies and long term studies on effects of various treatment modalities of primary lung LELC are limited. It has been reported that lung LELC has a statistically significant better prognosis than other NSCLCs [22,23]. Han et al found significant difference in survival rate of LELC compared with non-LELC in their retrospective cohort study. This is seen in the 2-year and 5-year survival rates of treated pulmonary LELC. The Stage III-IV disease survival numbers are high at 80.8% and 60.6% respectively (*P* < .05).

This case illustrates the primary treatment objective of advance stage LELC disease can be more than just improving quality of life and survival. With its better prognosis and prolonged stable disease state as seen in this case, selected patients with good functional status, if detected at an early stage, can be managed with curative intent.

Latent EBV infection of epithelial cells has been implicated in the pathogenesis of NPC. However, the carcinogenesis of LELC is still unclear and the association with EBV induced tumourigenesis is uncertain with the absence of association with EBV genome in the Caucasian LELC lung population and the low incidence of tobacco smoking history in this population of lung cancer [24]. Future studies can be done to explore the natural history of LELC, its metastatic behaviour and comparison be made to define the clinicopathologic or molecular characteristics of carcinoma from the various anatomical sites. Current studies of the oncogenesis of EBV infection can also be further explored, comparing the post treatment prognosis of EBV associated versus non-EBV associated LELC.

## Conclusion

We report of a case of metastatic LELC of the lungs which is a subtype of non small cell lung cancer. The association of LELC with EBV infection is still uncertain but the detection of viral DNA definitely facilitates diagnosis especially in the Asian population subgroup. It carries a better prognosis than any other type of NSCLCs. It is shown to be both radio and chemo sensitive. Good survival outcome can be attained even with stage IV disease, if treated aggressively on diagnosis with chemoradiation, supplemented with surgical resection. It is important to establish cross disciplinary collaboration in order to optimize the various treatment modalities of surgery, chemotherapy and radiotherapy, all available to treat LELC. This maximizes the patient’s lifespan bringing him/her to the next juncture where targeted cancer therapies become available and can be selectively adopted for further treatment of the primary cancer.

## Consent

Written informed consent was obtained from the patient for publication of this report and accompanying images. A copy of written consent is available for review if requested.

## Abbreviations

CT, Computed Tomography; EBV, Ebstein-barr Virus; LELC, Lymphoepithelial-like carcinoma; ISH, In-situ hybridization; NPC, Nasopharyngeal carcinoma; NSCLC, Non small cell lung cancer; WBRT, Whole brain radiotherapy.

## Competing interest

The authors declare that they have no competing interests.

## Authors’ contribution

WFSJ performed critical appraisal of the literature and wrote the manuscript. MTCC supervised, assisted in the critical appraisal of the included studies and editing of the manuscript. Both authors contributed to the final proof-reading of the manuscript.
